# Manufacturing and Financial Evaluation of Peptide-Based Neoantigen Cancer Vaccines for Triple-Negative Breast Cancer in the United Kingdom: Opportunities and Challenges

**DOI:** 10.3390/vaccines13020144

**Published:** 2025-01-29

**Authors:** Adriana Novakova, Stephen A. Morris, Ludovica Vaiarelli, Stefanie Frank

**Affiliations:** Department of Biochemical Engineering, University College London, Bernard Katz Building, Gower Street, London WC1E 6BT, UK; adriana.novakova.20@alumni.ucl.ac.uk (A.N.); stephen.morris@ucl.ac.uk (S.A.M.)

**Keywords:** neoantigen cancer vaccine, triple-negative breast cancer, cancer vaccine manufacturing, cost analysis, pricing strategy

## Abstract

This review evaluates the financial burden of current treatments for triple-negative breast cancer (TNBC) and projects potential financial scenarios to assess the feasibility of introducing a peptide-based neoantigen cancer vaccine (NCV) targeting the disease, using the UK as a healthcare system model. TNBC, the most aggressive breast cancer subtype, is associated with poor prognosis, worsened by the lack of personalised treatment options. Neoantigen cancer vaccine therapies present a personalised alternative with the potential to enhance T-cell responses independently of genetic factors, unlike approved immunotherapies for TNBC. Through a systematic literature review, the underlying science and manufacturing processes of NCVs are explored, the direct medical costs of existing TNBC treatments are enumerated, and two contrasting pricing scenarios for NCV clinical adoption are evaluated. The findings indicate that limited immunogenicity is the main scientific barrier to NCV clinical advancement, alongside production inefficiencies. Financial analysis shows that the UK spends approximately GBP 230 million annually on TNBC treatments, ranging from GBP 2200 to GBP 54,000 per patient. A best-case pricing model involving government-sponsored NCV therapy appears financially viable, while a worst-case, privately funded model exceeds the National Institute for Health and Care Excellence (NICE) cost thresholds. This study concludes that while NCVs show potential clinical benefits for TNBC, uncertainties about their standalone efficacy make their widespread adoption in the UK unlikely without further clinical research.

## 1. Introduction

Breast cancer represents the most commonly diagnosed cancer in the UK, with an annual incidence of 60,000 cases [1]. The disease is classified into four immunohistochemical subtypes based on receptor expression on the tumour surface. Among these, two express oestrogen and progesterone receptors, while the third subtype expresses human epidermal growth factor receptor 2 (HER2). In contrast, triple-negative breast cancer (TNBC), lacking the expression of hormone receptors, accounts for 15% of breast cancer cases and is known for its aggressive nature, resulting in a poorer prognosis [2]. Specifically, only 10% of metastatic TNBC patients survive beyond 5 years, compared to 30% in other metastatic breast cancer subtypes [3]. Furthermore, TNBC patients are twice as likely to develop metastases compared to non-TNBC subtypes [4]. Limited treatment options contribute to the dismal prognosis, with chemotherapy as the sole standardised approach. However, TNBC exhibits high immunogenicity, making it amenable to cancer immunotherapy strategies [5,6]. Despite the established role of immunotherapy in oncology, the development of TNBC-specific agents, like immune checkpoint inhibitors (ICIs), has been constrained by the complex and heterogeneous nature of TNBC tumours [7]. Consequently, attention has shifted to alternative immunotherapies, notably therapeutic cancer vaccines, aimed at augmenting tumour-specific immune responses [8]. Unlike conventional vaccines that establish prophylactic immune memory by administering pathogenic antigens, cancer vaccines seek to enhance the immune response against pre-existing tumour antigens within the body [9].

Several outcomes are needed to enable the successful rollout of new clinical interventions. The primary is an indication of clinical effectiveness by a clinical trial. But, it is also important to evaluate cost effectiveness and affordability to healthcare systems. The study of these aspects in parallel will help to enable a rapid rollout of clinically effective interventions.

As no personalised neoantigen cancer vaccine has been commercialised to date, this review offers a comprehensive outlook on the prospects and hurdles associated with the therapy.

The principal aim of this review is to analyse the physiological properties and the proposed manufacturing process of a peptide-based neoantigen cancer vaccine (NCV) for TNBC, identify the direct medical costs of TNBC treatments, and project two NCV pricing schemes using the UK as a model healthcare system. Our analysis shows that the potential clinical adoption of an NCV for TNBC is highly contingent upon the successful identification of key neoantigens and the definitive pricing model brought forward. We propose a multi-neoantigen setting with a TLR agonist that ought to be investigated for TNBC. Combination therapies may result in an opportunity to lower the current high TNBC therapy costs by leveraging the positive financial impact that an NCV could bring by enhancing clinical outcomes under such a therapeutic framework.

## 2. Immunotherapy Indication for Triple-Negative Breast Cancer

TNBC represents a group of heterogeneous tumours and currently lacks specialised treatment options, with chemotherapy representing the standard of care despite several limitations. It was reported that over 27% of breast cancer patients undergoing chemotherapy experience life-threatening adverse events, like severe diarrhoea and shortness of breath [1]. Moreover, TNBC patients frequently develop chemotherapy resistance, underscoring the critical need for novel therapeutic interventions. Prognostic outcomes for TNBC are mainly influenced by breast cancer stage (Table 1). Chemotherapy has been shown to be effective for TNBC in stages I and II, but stage III patients had an average survival time of 40 months, while metastatic patients had only 13 months [10,11] (for cancer stage overview, refer to Table 1). Pogoda et al. [12] observed a 35% recurrence rate within six years among metastatic TNBC patients initially diagnosed in stages II and III. These findings underscore the limited efficacy of chemotherapy in managing TNBC and emphasise the pressing need for targeted, personalised therapeutic strategies over generalised approaches.

**Figure 1:**
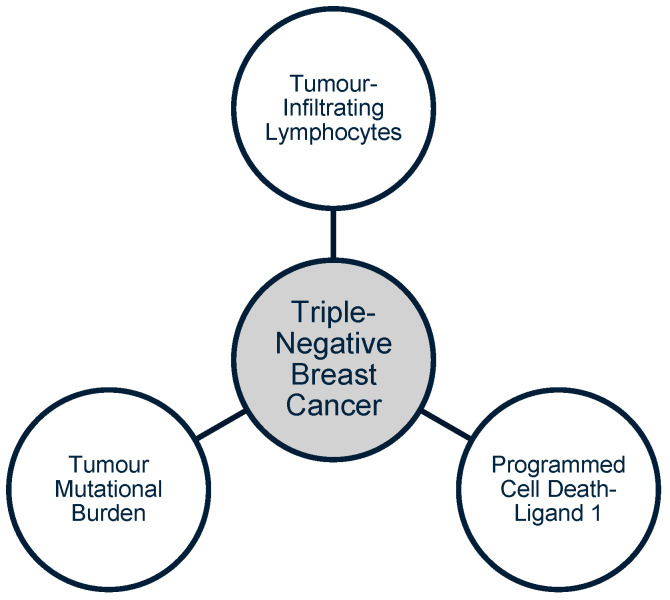
Implications for immunotherapy in TNBC treatment. These three TNBC features (white circles) allow for cancer immunotherapy agents.

Immunotherapy offers promise in customising TNBC therapies, particularly in relation to the distinct features of the tumour microenvironment. Compared to other breast cancer subtypes, TNBC exhibits higher levels of tumour-infiltrating lymphocytes (TILs), programmed cell death-ligand 1 (PD-L1) expression, and increased tumour mutational burden (TMB) [13] (refer to Figure 1).

Studies have demonstrated a strong association between higher levels of TILs and reduced metastasis [6,14,15], with low TIL levels correlating with higher relapse rates in stage I and II TNBC patients [15]. PD-L1, a protein overexpressed on cells that binds to programmed death protein 1 (PD-1) on immune cells, plays a crucial role in stimulating immune responses. High PD-L1 expression is strongly correlated with improved survival rates in TNBC patients, with TNBC exhibiting the highest PD-L1 expression among all breast cancer subtypes [13,16,17]. Additionally, TNBC’s TMB, reflecting the number of antigens presented to immune cells, further drives the exploration of immunotherapy applications. Numerous studies have demonstrated that TNBC cases with high TMB derive greater benefits from immunotherapy [18,19]. The described features of the TNBC tumour microenvironment highlight the significance of immunotherapy in tailoring TNBC therapy options.

Presently, approved immunotherapy agents for TNBC include immune checkpoint inhibitors (ICIs), atezolizumab, and pembrolizumab. For a comprehensive review of ICI therapy for TNBC, refer to Keenan and Tolaney [20]. Atezolizumab is indicated for administration alongside chemotherapy in stage III and IV TNBC, while pembrolizumab, also approved for combination therapy, is prescribed for stage II and III disease [21,22]. Patient eligibility for either agent depends on PD-L1 expression status in TILs. However, studies have shown varying results regarding the prevalence of PD-L1 expression in TNBC patients, ranging from 21% to 53% [23,24]. While the limited patient pool poses a significant constraint on targeted ICI therapy, it also presents an opportunity for alternative cancer immunotherapies that are not reliant on genetic predisposition, such as therapeutic cancer vaccines.

## 3. Neoantigen Cancer Vaccines

Tumour cells express two types of peptide antigens capable of triggering an immune response: tumour-associated antigens (TAAs) and mutation-derived antigens (neoantigens). TAAs, originating from overexpressed normal cellular proteins, are self-antigens aberrantly expressed in multiple patients [25]. In contrast, neoantigens are patient-specific non-self antigens resulting from unique genetic alterations not shared among patients [26]. Neoantigens are immunogenic peptides representing the key component of peptide-based neoantigen cancer vaccines (NCVs).

Current cancer vaccines are classified into four categories: peptide based, cell based, virus based, and nucleic acid based (Table 2). While all aim to enhance the cancer-immunity cycle (CIC) (refer to Liu et al. [26] for a detailed CIC description), they differ in antigen presentation, irrespective of antigen type. Peptide-based vaccines directly deliver antigen epitopes, initiating the CIC through APC processing. Despite the challenge of weak immunogenicity of peptide-based vaccines, the platform is favoured in research and development (R&D) due to its straightforward manufacturing process correlating with lower vaccine costs [27] and potential for optimisation through the incorporation of adjuvants, offering a practical approach to cancer immunotherapy. Neoantigens formulated in peptide-based cancer vaccines could offer a personalised approach to improve the survival rates of TNBC patients. While there is considerable industry interest in such vaccines, most studies are still in the early stages, with two ongoing investigations focusing on peptide-based NCVs for TNBC (Table A1). However, this platform faces significant challenges in terms of poor immunogenicity, often resulting from the inherently small size of neoantigens [28].

### 3.1. Research and Methodology

Research and methodology are organised into three sections: (1) a literature review of the underlying science of peptide-based neoantigen cancer vaccines (NCVs), (2) direct medical cost calculation of TNBC treatment in the UK, and (3) the evaluation of two pricing strategies for the clinical adoption of NCVs in treating this cancer subtype.

### 3.2. Literature Search

**Figure 2:**
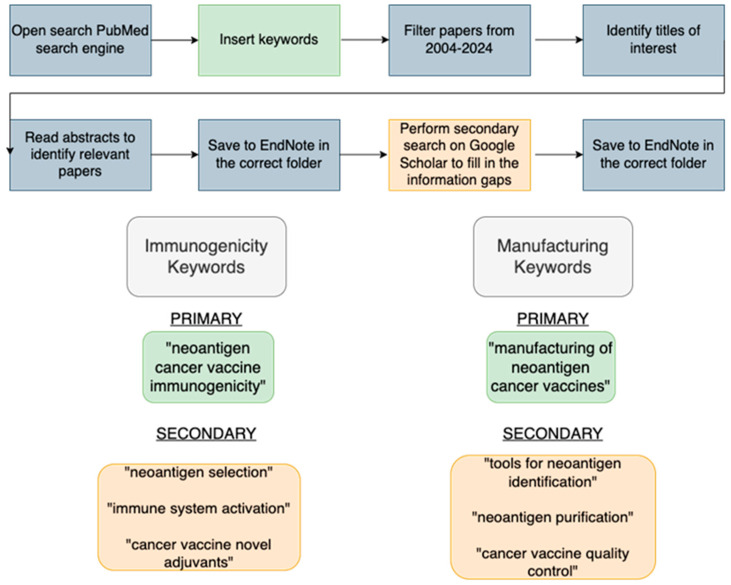
Literature search process and primary and secondary keywords. This method was used to perform a systematic literature review in “Immunogenicity of Neoantigens” and “Proposed Manufacturing of Neoantigen Cancer Vaccines”.

The literature search process shown in Figure 2 was conducted using PubMed and Google Scholar. PubMed was chosen to screen research papers and pre-clinical and clinical study reports, providing a comprehensive view of the neoantigen cancer vaccine (NCV) landscape, and Google Scholar was used to fill in information gaps. EndNote Library was instrumental in categorising publications into folders. The search inclusion criteria were the publication period (2004–2024) and the incorporation of keywords in the titles. The most important primary and secondary keywords used for the search are shown in Figure 2. Any papers published before 2004 were excluded from the review due to the fast-paced nature of the research area. Only peer-reviewed publications were included in this study, and manual quality control checks were carried out to ensure the reliability of data. Clinical trial data were sourced from ClinicalTrials.gov, verifying sources, sponsors, and eligibility criteria to ensure unbiased data. All clinical trial study identification numbers are listed in Table A1.

### 3.3. Direct Medical Cost Data Extraction

**Figure 3:**
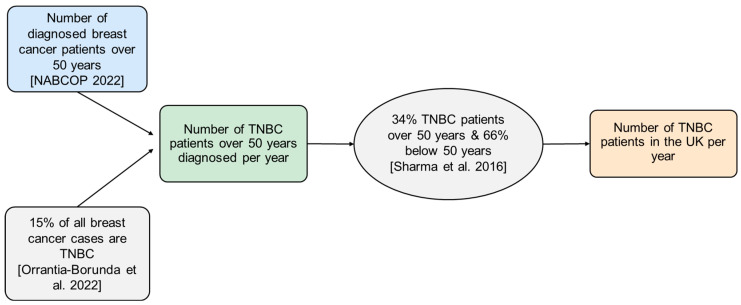
Method for the calculation of annual TNBC patients diagnosed in the UK. Assumptions outlined in the figure were used to calculate the total annual number of TNBC patients [4,34,35].

**Figure 4:**
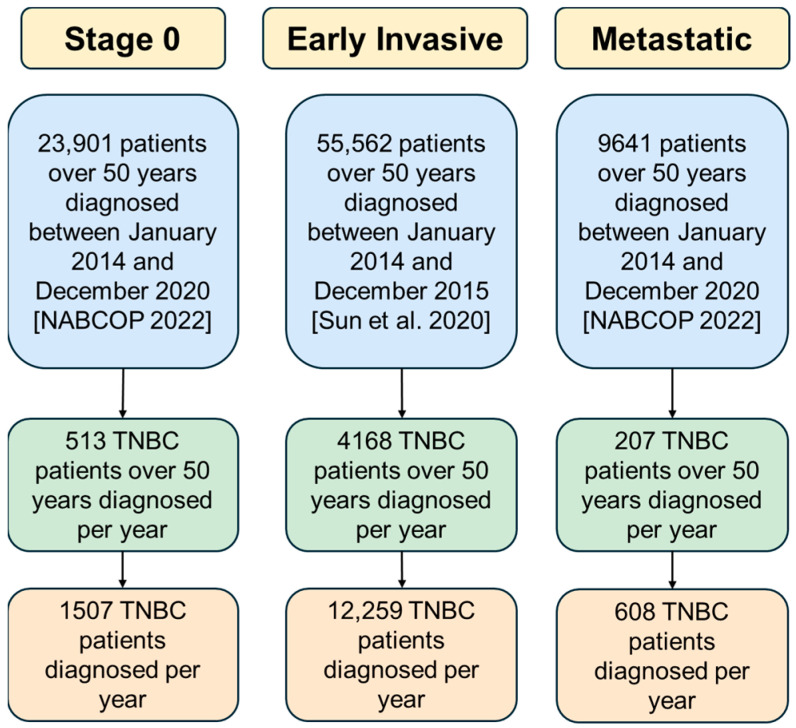
Annual distribution of TNBC patients per cancer stage. The calculation method is applied from Figure 3. The numbers of patients per breast cancer stage were directly derived from the cited sources [35,36]. Stage 0 and metastatic stage patients were adjusted to an annual number of new breast cancer stages, and the values were further adjusted to extract the TNBC proportion of patients. The final number accounted for TNBC patients below 50 years since the primary data did not contain these patients. Notably, the data used to quantify patients in the early-invasive subgroup are limited to the year 2015, compared to more recent data available to quantify patients in stage 0 and metastatic subgroups. This implies a limited degree of accuracy of the estimated numbers.

The first step in calculating direct medical costs was to determine the annual number of TNBC patients diagnosed in the UK, as shown in Figure 3. Data from the National Audit of Breast Cancer Patients (NABCOP) served as the primary source to quantify patients over 50 years, assuming that 15% of all breast cancer cases were TNBC [34,35]. Numbers of stage 0 and metastatic stage breast cancer patients were directly derived from the NABCOP report. The figures were further adjusted to include younger women, considering TNBC’s higher prevalence among this demographic, which was not covered in the NABCOP data. A study reporting that only 34% of TNBC patients are above 50 years old was adopted as the status quo to calculate the total number of TNBC patients [4]. The application of Figure 3 is demonstrated in Figure 4, illustrating the distribution across the cancer stages.

#### 3.3.1. Conventional Therapies

**Figure 5:**
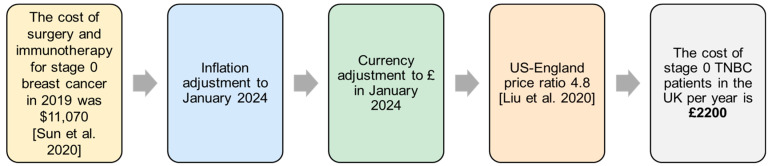
Method for calculating the cost per stage 0 TNBC patient in the UK. This approach was taken due to a lack of stage 0 therapy cost information in the UK [36,37].

The costs of conventional therapies for each cancer stage were calculated using various methodologies. Note that all subsequent costs were adjusted to January 2024 Great British Pounds using the UK and US Consumer Price Index. Since no data on stage 0 therapy procedures are publicly reported within the NHS, we calculated the cost per patient following the rationale outlined in Figure 5. An equal distribution between stages I to III was then assumed for the early-invasive stage. Lastly, the known proportions of patients receiving treatment per stage were used to identify the real number of patients requiring treatment presented in Table 3. However, limitations to the process are associated with the assumptions made, as these numbers are not provided by the NHS.

The cost values discussed in the financial burden of the triple-negative breast cancer section were adopted from the literature as outlined in Table 4.

#### 3.3.2. Immune Checkpoint Inhibitor Therapy

The direct medical costs for treatment regimens with atezolizumab and pembrolizumab were obtained from publicly available documents (Table 5). Additionally, the number of patients eligible for immune checkpoint inhibitor therapy depends on programmed cell death-ligand 1 expression levels in tumour-infiltrating lymphocytes, with an assumed calculated mean of 37% representing the range of 21% to 53%, as reported in studies [23,24] (Table 6).

### 3.4. Pricing Scenarios

An identical vaccine formulation and regimen, outlined in Table 7, was assumed for two pricing scenarios—the best-case decentralised government-sponsored therapy model and the worst-case privately owned therapy model with in-house production.

**Figure 6:**
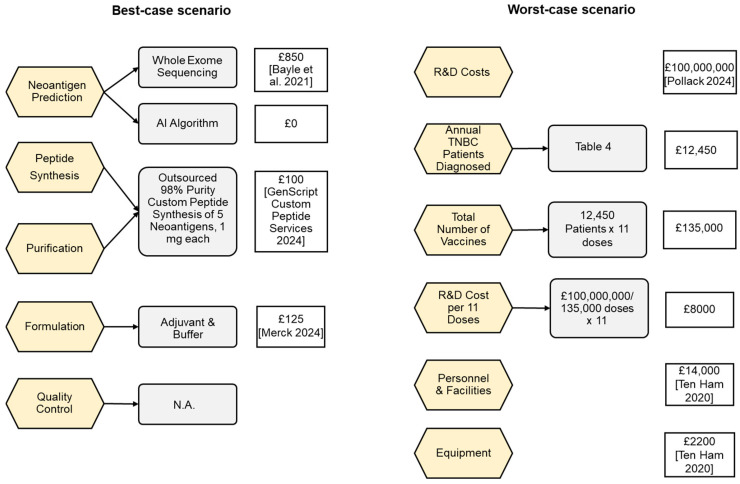
Cost per manufacturing step of the best-case scenario (**left**) and worst-case scenario costs (**right**). The left figure follows the manufacturing process proposed in this review [46,47,48].The right figure shows a calculation based on assumptions of heavy investments and mirroring costs outlined for another personalised therapy [32,49]. The value is associated with major assumptions due to the novelty of NCVs. Both costs represent the total cost per patient, as detailed in Table 7. Quality control is not applicable for the best-case scenario based on the assumption that the neoantigens have been tested in production and this cost is included in the previous steps.

Figure 6 illustrates the best-case scenario which assumes the same process steps as discussed in the section “Proposed Manufacturing of Neoantigen Cancer Vaccines”, and the worst-case scenario, which assumes additional costs to recover initial investments.

## 4. Results and Discussion

### 4.1. Triple-Negative Breast Cancer Vaccine Clinical Trials

**Figure 7:**
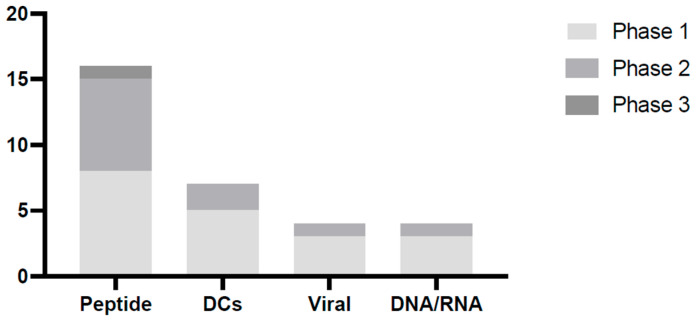
Breakdown of TNBC cancer vaccine platforms. The analysis demonstrates the significant industry interest in peptide-based cancer vaccines. The data are detailed in Table A1. Created from clinicaltrials.gov.

Following an analysis of the different types of cancer vaccines for triple-negative breast cancer (TNBC), it was decided to prioritise peptide-based neoantigen cancer vaccines (NCVs). The data presented in Appendix A, summarised in the accompanying Figure 7, support this decision, highlighting the prevalence of ongoing clinical trials investigating peptide-based vaccines in the context of TNBC. Furthermore, TNBC tumours compared to other cancer types have a high mutational burden, resulting in large proportions of individual mutations (neoantigens). Therefore, the focus of this paper is on neoantigen formulations rather than tumour-associated cancer vaccines [50,51].

### 4.2. Immunogenicity of Neoantigens

#### 4.2.1. Physiological Properties of Neoantigens

Neoantigens are typically categorised into short and long peptides based on their structural characteristics. Short peptides, however, often fail to induce an anti-tumour effect [52]. In contrast, long peptides, ranging from 13 to 18 amino acids, trigger both cytotoxic T-cells and CD4+ immune responses. This dual activation is attributed to the internalisation of long peptides by DCs, followed by presentation via both MHC I and II molecules, thereby bypassing immune tolerance mechanisms [53]. Synthetic long peptides (SLPs) are noteworthy for their ability to induce robust immune responses, including cytokine production and direct tumour elimination by CD4+ T-cells. Research highlights the pivotal role of CD4+ T-cells in initiating neoantigen-specific T-cell responses, emphasising the importance of utilising SLPs in cancer vaccine formulations [54]. These findings underscore the importance of researching SLPs to prolong antigen cross-presentation, thereby enhancing anti-tumour immune responses.

#### 4.2.2. Vaccine Formulation Solutions

Further immunogenicity enhancement can be achieved by incorporating immunostimulatory adjuvants. Among the most frequently utilised agents are toll-like receptor (TLR) agonists, with polyinosinic-polycytidylic acid-poly-l-lysine carboxymethylcellulose (poly-ICLC) employed in a significant number of TNBC peptide-based NCV clinical trials highlighted in Figure 7. Studies have demonstrated that poly-ICLC can evoke robust T-cell and antibody responses, while also facilitating DC maturation and enhancing the anti-tumour efficacy of cancer vaccines [55,56].

Enhancing the immunogenicity of NCVs can also be achieved by incorporating multiple neoantigens into a single vaccine. Zhang, et al. [57] demonstrated the effectiveness of this method by successfully incorporating 11 neoantigens per vaccine per patient, resulting in a durable immune response and validating the viability of this approach. Moreover, employing a multi-neoantigen strategy promotes epitope spreading, wherein additional neoantigen-specific T-cells are generated against neoantigens not contained within the vaccine [58]. This approach shows promise in addressing the poor prognosis associated with high tumour heterogeneity in TNBC, as the expanded immune landscape may facilitate overcoming this challenge [59].

These findings outline the potential neoantigen cancer vaccine (NCV) formulation, i.e., a multi-neoantigen setting with poly-ICLC adjuvant, used to project pricing scenarios later in the review.

**Figure 8:**
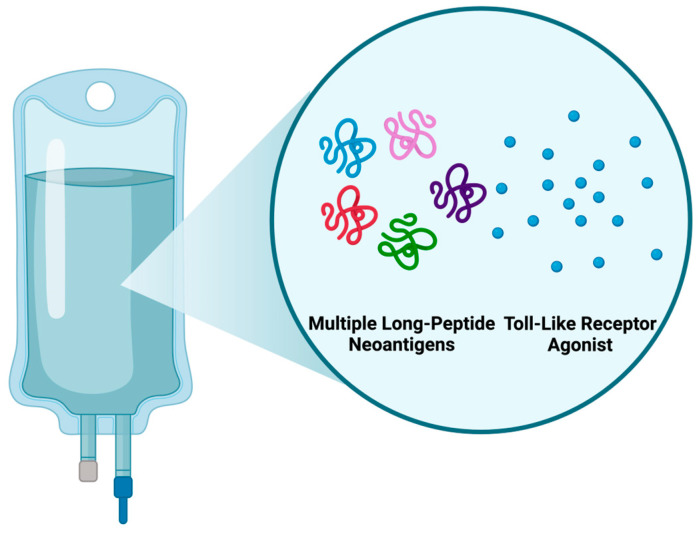
Recommended formulation of an NCV. To address NCVs’ low immunogenicity, a formulation with multiple neoantigens and adjuvants as immunostimulators and delivery systems is proposed. Created with Biorender.

While peptide-based NCVs are a promising approach, their limited immunogenicity poses a challenge compared to other platforms. This can be addressed by incorporating immunogenic long-peptide neoantigens with adjuvants in a multi-neoantigen setup (Figure 8). This strategy extends neoantigen presentation on DCs, stimulating robust CD8+ and CD4+ T-cell responses and enhancing overall immune activation [54]. Incorporating toll-like receptor (TLR) agonists as adjuvants is recommended to stimulate the immune response; hence, poly-ICLC (TLR agonist) will be used in projecting the following NCV pricing scenarios.

### 4.3. Proposed Manufacturing of Neoantigen Cancer Vaccines

**Figure 9:**
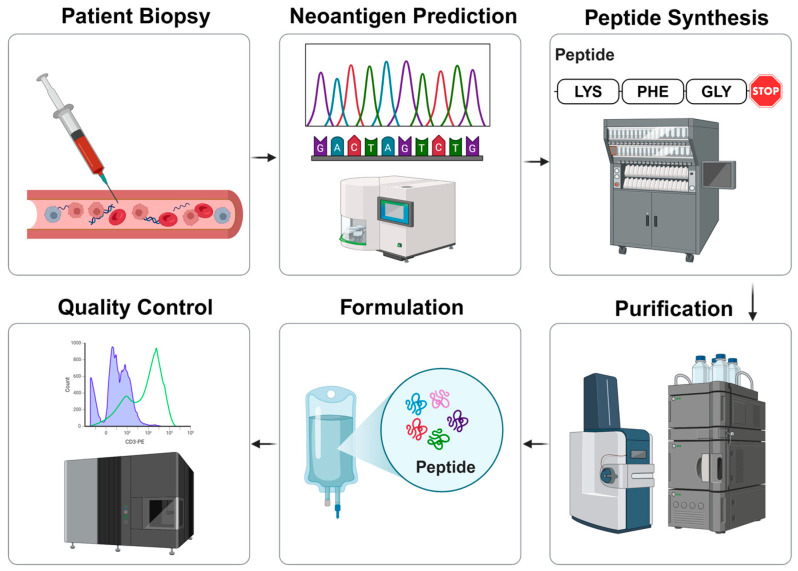
The proposed manufacturing process of an NCV. The process begins with obtaining a patient sample to predict immunogenic neoantigens and the arrows show the subsequent steps. The first step can utilise next-generation sequencing (NGS) tools, including whole exome sequencing, and validate the results by HLA typing, peptide processing, and peptide-MHC binding prediction. Next, the identified neoantigens synthesis will be outsourced to protein synthesising companies, significantly driving the low-cost dynamics of the process. The peptide purification will be outsourced as well, and the neoantigens will be delivered with 98% purity [47]. The proposed formulation of an NCV is an intravenous injection to stimulate a strong anti-tumour response. The quality control (QC) for an NCV will determine safety, immunogenicity, and efficacy by performing immunogenic assays and analysing adverse events and standard oncology endpoints. Created with Biorender.

Building on the findings from the previous section, an ideal TNBC NCV should feature multiple accurately identified neoantigens with high-affinity binding to HLA class II, determined through next-generation sequencing (NGS) methods. Challenges persist in identifying highly immunogenic and soluble neoantigens, necessitating further development of bioinformatic tools. Established and scalable approaches to neoantigen synthesis positively impact NCV production time. Intravenous administration of the vaccine is recommended, but further research is needed to determine optimal dosage and vaccination schedules. Quality controls (QCs) encompass safety, immunogenicity, and efficacy screening and testing, employing standard oncology endpoints and analytical assays. Overall, NCV manufacturing presents a viable aspect of launching NCVs for TNBC (illustrated in Figure 9), yet further advancements are necessary in neoantigen prediction, formulation, and QC processes due to the early-stage R&D of NCVs.

#### 4.3.1. Neoantigen Prediction

The initial phase in selecting optimal neoantigens for an NCV involves neoantigen identification, commencing with patient biopsy and tissue analysis through comparative assessment of single nucleotide variants (SNVs) between tumour and non-tumour DNA [60]. Clinical evidence shows that SNV-derived neoantigens in NCVs can induce disease regression and enhance T-cell responses [61]. Despite various genome analysis toolkits available, like whole exome sequencing, a manual review of matched tumour-normal samples is recommended due to screening tool limitations [62], potentially prolonging the turnaround time from biopsy to vaccine formulation. Research indicates multiple possible neoantigen categories, including tumour-specific fusion proteins resulting from gene translocations, which exhibit considerable genetic instability within chromosomes and can generate highly immunogenic neoantigens [63]. While research on fusion genes in TNBC is limited, the available data appear promising. Wang et al. [64] identified 22 recurring fusion proteins in TNBC patients, identifying a novel fusion biomarker. Although no other fusion proteins have been identified for TNBC, research on fusion neoantigens in other cancers has shown their ability to enhance T-cell responses and eliminate tumour cells [65]. Therefore, while additional validation of genome analysis toolkits is necessary, these findings highlight encouraging strategies for clinical application, as successfully demonstrated in the TNBC context.

Validation steps in neoantigen prediction include HLA typing, peptide processing, and peptide–MHC binding prediction. Precise HLA identification is crucial due to MHC molecule polymorphism, with over 12,000 allele modifications [66]. While next-generation sequencing (NGS) has achieved high accuracy for MHC class I, further development for class II HLA sequencing is required [67,68]. Peptide processing prediction focuses on the binding affinity between the patient’s MHC molecule and the given neoantigen, with challenges, such as peptide cleavage, potentially hindering successful peptide loading onto APC [69,70]. MHC binding prediction, based on HLA typing, is considered the most selective step but results in inaccurate neoantigen predictions due to factors such as peptide immunogenicity and gaps in HLA allele datasets [68,71]. Only 20% of in silico-predicted neoantigens exhibited immunogenicity, and 55% of in vitro-predicted immunogenic neoantigens elicited an immune response after binding to HLA [72,73]. One strategy to address these challenges involves a scoring system that considers factors, including HLA mutation frequencies, neoantigen transcription abundance, and MHC binding likelihood, showing a 95% accuracy in predicting the immunogenicity of selected neoantigens [74]. Developing methods for accurately identifying the most immunogenic neoantigens is imperative, given an average of 60 neoantigen mutations per patient [73]. These findings suggest that bioinformatic tools can accurately predict neoantigens, but additional validation of the methods is needed to establish the process.

#### 4.3.2. Peptide Synthesis

Upon completing the neoantigen identification, two primary methods are available for neoantigen production: peptide synthesis and genetic engineering. Peptide synthesis offers distinct advantages, notably in terms of cost effectiveness and production efficiency [75]. This process relies on solid-phase peptide synthesis (SPPS), which involves the sequential addition of amino acids to the growing peptide chain [76]. Various companies offer peptide synthesis at remarkably low costs, with prices typically in the region of GBP 19 per 4 mg of a peptide [47]. This affordability significantly influences the cost dynamics of NCV manufacturing. Mijalis et al. [77] demonstrated an automated SPPS approach capable of producing an amino acid residue in 40 s. Considering that neoantigens should preferably be SLPs ranging from 13 to 18 amino acids in length, the production time for a single neoantigen is approximately 12 min. Consequently, producing the required amount of a neoantigen can only take several hours. Given a concurrent neoantigen synthesis for a multi-neoantigen vaccine, the production time would not increase. Thus, peptide synthesis represents a financially feasible and scalable approach for manufacturing NCVs.

#### 4.3.3. Purification

Once the neoantigen synthesis is completed, purification proceeds via high-performance liquid chromatography (HPLC). Mass spectrometry is commonly utilised to confirm the neoantigen structure and has demonstrated accurate sensitivity. Reversed-phase HPLC has proven effective in eliminating impurities post-neoantigen synthesis, often in combination with ion exchange or gel filtration chromatography [76,78]. However, a challenge in purifying SLPs arises from their amino acid sequence insolubility [68]. While studies have not extensively addressed this issue in neoantigens as SLPs, temporary peptide tags have demonstrated success in enhancing the solubility of other types of SLPs [79,80,81]. Additionally, computational tools for neoantigen prediction aim to address the purification challenges by identifying hydrophobic sequences. These tools help select the most viable neoantigen residues, serving as a decision-making tool for correct neoantigen manufacture [82]. However, neoantigens fall within the standard frame for peptide synthesis companies, which often provide peptides at 98% purity, streamlining the purification step in the manufacturing process [47]. These findings suggest that challenges related to neoantigen purification warrant further investigation; however, neoantigens as SLPs can achieve clinical-grade purity through outsourcing to peptide synthesis companies, eliminating the challenge.

#### 4.3.4. Formulation

Although the formulation of NCVs lacks standardisation, several clinically established considerations exist. Intravenous administration of NCVs has demonstrated a stronger immune response compared to subcutaneous routes, with subcutaneous injection leading to T-cell deletion via chronic T-cell stimulation, resulting in low anti-tumour responses in clinical trials [83]. Moreover, proper stimulation timing and adjuvant selection are crucial to induce T-cell response alongside appropriate costimulation and cytokine signalling. Achieving timely APC maturation alongside neoantigen delivery is also essential for eliciting a robust anti-tumour response [75]. Another challenge in NCV manufacture is the turnaround time from biopsy to finalising the vaccine formulation, which typically takes 3–4 months [61]. This extended period may exceed the life expectancy of TNBC patients, necessitating further investigation into correct intravenous NCV formulation, along with a pressing need to shorten the vaccine production time.

#### 4.3.5. Quality Control

**Figure 10:**
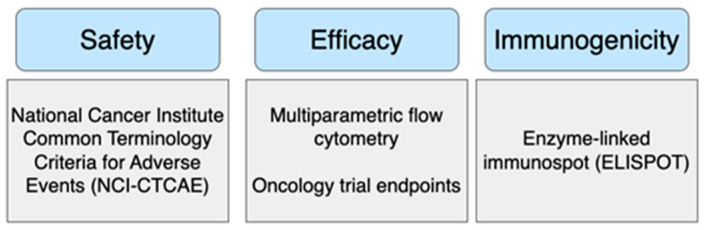
Quality control (QC) requirements for a safe, effective, and immunogenic NCV. These QCs were adopted from the two NCVs for TNBC trials (Section 3.1), demonstrating industry-accepted controls.

Industry-standard quality controls (QCs) for NCVs include safety, immunogenicity, and efficacy (Figure 10). Both trials employ the National Cancer Institute Common Terminology Criteria for Adverse Events (NCI-CTCAE) to assess safety, while efficacy and immunogenicity are evaluated using enzyme-linked immunospot (ELISPOT) analyses, multiparametric flow cytometry, and standard oncology trial endpoints, such as the clinical response rate and overall survival [84,85]. ELISPOT assays objectively assess NCV immunogenicity by quantifying the frequency of immune cells producing specific cytokines linked to tumour-specific immune responses, validated for neoantigen-specific T-cell responses in cancer patients [86]. Multiparametric flow cytometry assesses cytokine production and the frequency of neoantigen-specific T-cells, providing a comprehensive view of the cancer vaccine efficacy [87]. The clinical response rate indicates the proportion of patients with tumour shrinkage or disappearance, reflecting vaccine efficacy, while overall survival directly reflects the vaccine’s impact on patients’ lives [88]. Thus, the analysis of the two NCV trials justifies current QC practices, providing authentic assessments of NCV safety, efficacy, and immunogenicity.

These findings suggest the overall viability of manufacturing NCVs. As a result, the following sections will focus on two main objectives: firstly, calculating the economic implications of managing TNBC in the UK using current therapies, and secondly, conducting a financial analysis of two different pricing scenarios for NCVs to assess their potential introduction to the UK market.

### 4.4. Financial Burden of Triple-Negative Breast Cancer

The cost of TNBC holds significant relevance due to the substantial resource utilisation associated with each TNBC stage, ranging from GBP 2200 to GBP 54,000 annually per patient, amounting to GBP 230,000,000 annually for the NHS. While attempts have been made to calculate the cost of TNBC in various countries [89], no studies have yet estimated the cost of TNBC therapies for the NHS. Although TNBC is associated with indirect costs, such as productivity losses and reduced quality of life, this review focuses solely on calculating the direct medical costs. Despite the TNBC financial burden being significant, it remains a question whether an NCV could alleviate direct costs. A potential for cost reduction at the advanced stages is in reducing the chemotherapy cycles and the regimen of immune checkpoint inhibitors.

#### 4.4.1. Direct Medical Costs of Conventional Therapies in the United Kingdom

**Figure 11:**
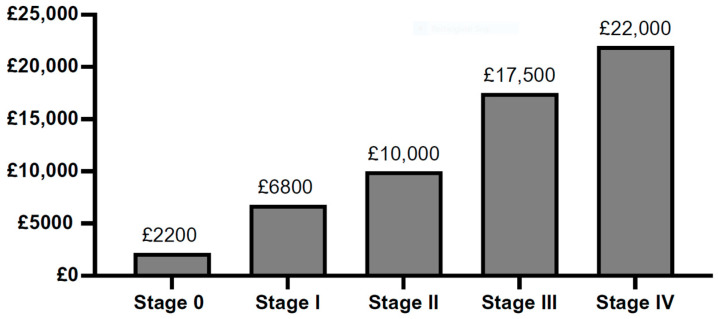
Annual direct medical costs of conventional therapies per patient in the United Kingdom. This figure demonstrates the cost of surgery, chemotherapy, and radiotherapy per TNBC stage, with references outlined in Table 4. These data were consolidated from various sources (shown in Table 4) due to a lack of economical appraisal of TNBC therapy costs in the United Kingdom.

The calculated cost of conventional therapy ranges from GBP 2200 to GBP 22,000 per patient annually, depending on the TNBC stage, as demonstrated in Figure 11. Evaluation of these numbers against reviewed figures is challenging due to a lack of available data. The most comparable study reported a mean cumulative 15-month cost of GBP 12,595 per breast cancer patient in the UK, equivalent to GBP 14,000 as of January 2024 [90]. Notably, the study was conducted before ICIs were available for TNBC treatment, so the reported value only considered the conventional therapies. For comparison, dividing the total yearly cost by the number of patients per stage in this review yields GBP 10,400 per patient. The difference between GBP 14,000 and GBP 10,400 may be due to more costly treatment options available for other breast cancer subtypes, potentially inflating the reported mean value of the published study. Numerous limitations are inherent in estimating the financial burden of TNBC, particularly considering that therapies may extend beyond a year, which is not factored into the calculations within this review. Additionally, TNBC is more common among younger women, which might lead to higher therapy rates than those shared by NABCOP [35], as NABCOP accounted for only women over 50 years who might not be offered the TNBC treatment due to other factors (age and/or pre-existing health conditions).

**Figure 12:**
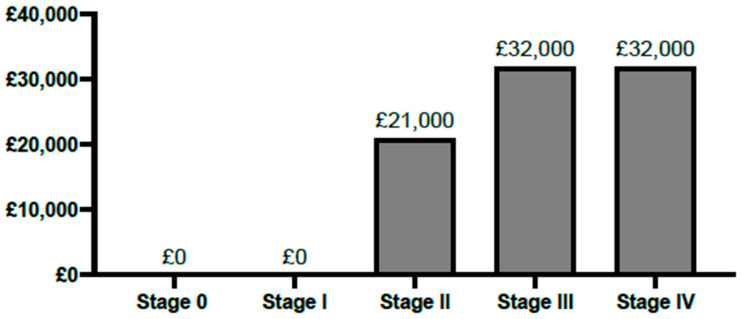
Annual direct medical costs of immune checkpoint inhibitor (ICI) therapies per patient in the United Kingdom. As no ICI therapy is available for stage 0 and I TNBC patients, the figure displays the annual cost of pembrolizumab per stage II TNBC patient. The stage III value represents the atezolizumab cost, although both ICI agents are available to stage III TNBC patients. Stage IV represents the atezolizumab cost.

The calculated costs for immune checkpoint inhibitor (ICI) therapy are shown in Figure 12. Annual pembrolizumab cost per patient was calculated to amount to GBP 21,000 and atezolizumab to GBP 32,000. The ICI therapy cost calculations are more precise due to available access to official reports, though they are likely overestimated due to discounts confidentially given to the NHS. Furthermore, as both ICI agents are available for stage III TNBC, the more expensive treatment (atezolizumab) was assumed to project the highest possible costs.

#### 4.4.2. Analysis of All Direct Medical Costs

**Figure 13:**
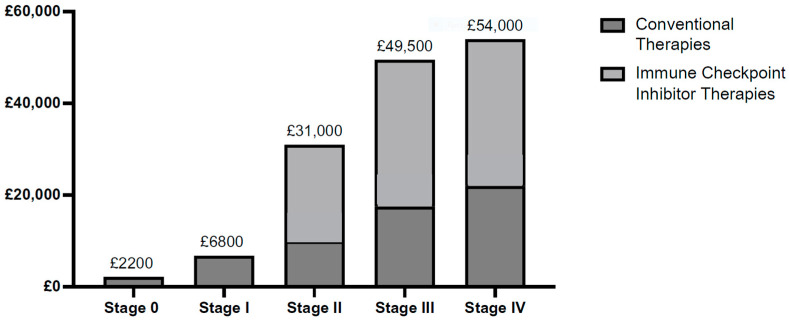
Yearly per-patient cost comparison per stage. Although both ICIs are available to stage III patients, the highest potential costs were assumed; therefore, atezolizumab was included (cost comparison is shown in Table 5). The cost of conventional therapies was determined from papers, aside from stage 0, which was calculated as per Figure 5. ICIs were calculated from regimen specifications from official reports (Table 5).

The costs vary significantly across different disease progression stages, as shown in Figure 13, with notable escalations in advanced disease stages. Stage 0 and I TNBC patients typically undergo surgery, with additional chemotherapy and radiotherapy for stage I. Moreover, the shorter therapy cycles also contribute to lower associated costs [36]. Costs markedly escalate from stage II onwards due to the inclusion of ICIs, although not all patients meet the eligibility criteria for these therapies. According to the assumptions in this paper, nearly two-thirds of patients do not express PD-L1, thus receiving only conventional treatment. However, the per-patient costs shown in Figure 13 (ranging from GBP 2200 to GBP 54,000) represent the maximum potential costs per stage, including ICIs. Interestingly, costs between stages III and IV are relatively comparable (GBP 49,500 and GBP 54,000). While stage IV TNBC patients have a median survival time of 13 months, the reported overall survival time for stage III patients is 92 months [10,91]. Therefore, the substantial costs of stage IV therapy and the reduced quality of life resulting from chemotherapy may contribute to the low therapy uptake, with only 25% of stage IV patients receiving any form of treatment, as shown in Table 3 [35].

**Figure 14:**
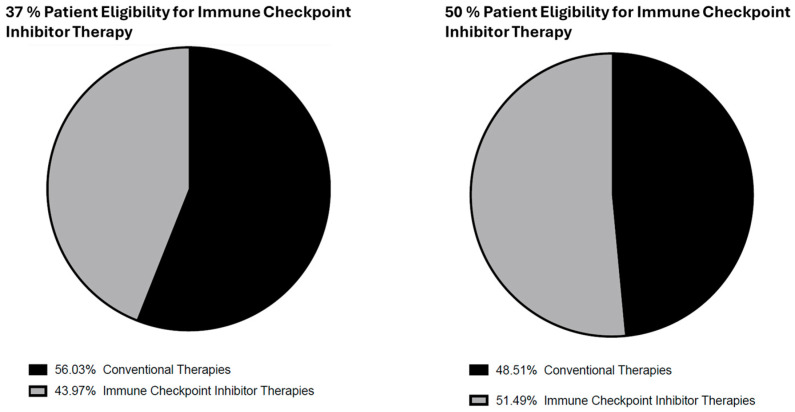
Annual cost breakdown of treating TNBC in the UK assuming an immune checkpoint inhibitor patient eligibility of 37% (**left**) and 50% (**right**). The left-hand graph demonstrates the total cost, which equals GBP 230,000,000 annually, with conventional therapies amounting to GBP 130,000,000 and immune checkpoint inhibitor therapies to GBP 102,000,000. The right-hand graph demonstrates the total annual cost of GBP 268,000,000. The majority of the costs are now associated with immune checkpoint inhibitor therapies (GBP 138,000,000). Data are shown in Table A2, Table A3 and Table A4.

The annual expenditure for treating all TNBC patients in the UK is projected to reach GBP 230,000,000, with conventional therapies representing GBP 130,000,000 (Table A2), and the proportions are shown in Figure 14. The ICI therapy based on 37% patient eligibility criteria was calculated to amount to GBP 102,000,000 (Table A3), representing the minority of annual TNBC therapy costs. Another financial evaluation was performed, which considered a 50% ICI eligibility of TNBC patients. As shown in Figure 14, this leads to a significantly higher value of ICI therapy costs, estimated at GBP 138,000,000 (Table A4). In this case, the ICIs represent most of the annual expenditure of TNBC therapy, which increases to GBP 268,000,000. These data imply that it is essential to identify the correct number of eligible patients.

It can be expected that an NCV would not eliminate existing therapy options but rather serve as an additional option to control the disease more effectively. For this reason, it is advised that NCV research focuses on the introduction of NCVs to TNBC stage II onwards. This could allow for cost alleviations, e.g., by reducing the number of ICI cycles or decreasing the frequency of ICI administration. Notably, an NCV could also reduce the need for intensive chemotherapy cycles. Although the chemotherapy costs are not heavily resource intensive, it could still result in improved therapy tolerability of patients and improve their quality of life.

The upcoming section will examine the costs of conventional therapies and ICIs in comparison to two hypothetical pricing models of a TNBC NCV. The analysis will aim to assess the potential feasibility of introducing the vaccine in the UK and its potential impact on reducing TNBC treatment costs in the country.

### 4.5. Pricing

#### 4.5.1. Incremental Cost Effectiveness Ratios of Approved Therapies

The incremental cost effectiveness ratio (ICER) provides insights into the cost effectiveness of different treatments in terms of life years gained due to the medication. According to the literature, the National Institute for Health and Care Excellence (NICE) has established a willingness-to-pay therapy threshold of GBP 50,000 per life year gained [92]. Hence, the current TNBC therapies were evaluated against this upper cost limit, as shown in Table 8. All ICER calculations were found to be below the threshold, considering that ICI therapies received a discount for NICE, resulting in even lower costs than depicted in Table 8. Notably, pembrolizumab exhibited the lowest ICER, although the median progression-free survival (PFS) is not officially confirmed by the manufacturer, given the recent approval of the therapy and the confidentiality surrounding its details. Therefore, the GBP 50,000 NICE threshold offers a constructive benchmark for evaluating the two hypothetical NCV pricing scenarios.

#### 4.5.2. Pricing Scenarios Assuming a Commercialisation of a Peptide-Based Neoantigen Cancer Vaccine for Triple-Negative Breast Cancer

Should clinical trial studies indicate the usefulness of NCVs, it will be essential to have models of how these treatments could be effectively implemented by healthcare systems, such as the UK-NHS. To this end, a best-case and a worst-case scenario were proposed and analysed to assess the feasibility of a hypothetical NCV. The assumption adopted for this section is a demonstrated efficacy of the NCV in phase III trials with limited side effects that are inferior to the benefits generated from the commercialisation of the NCV. It is important to assume such a scenario due to the currently early phase but promising research about NCVs for TNBC and to allow for industry preparedness ahead of a real-life NCV launch. The proposed NCV formulation outlined in Table 7 was applied to both pricing scenarios, involving a multi-neoantigen vaccine administered in 11 doses with a PFS of 5.6 months. In the best-case scenario, treatment was assumed to be administered at cost with a decentralised approach and zero intermediary supply chain. All steps were assumed to be outsourced, following the proposed manufacturing process described in this review in Section 4.3 and shown in Figure 9. Vaccine delivery would be facilitated at existing administration locations, such as hospitals. It was further assumed that no company owned the vaccine, and no profits were generated. This approach resulted in a cost of GBP 1100 per patient, with an ICER of GBP 2400, as shown in Table 9. Conversely, the worst-case scenario assumed a privately funded NCV aimed to recover estimated initial R&D costs and the establishment of an in-house manufacturing site, albeit without any profit margin. The total cost per year amounted to GBP 25,000, resulting in an ICER of GBP 55,000, as outlined in Table 9.

**Figure 15:**
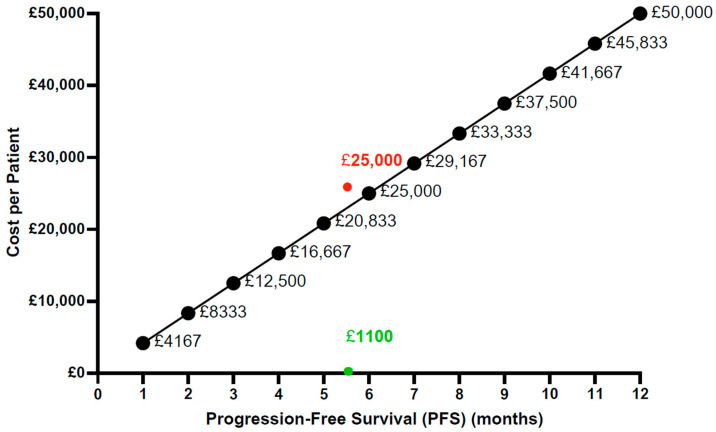
Cost scenarios plotted against the NICE ICER threshold in a hypothetical scenario of the commercialisation of an NCV for TNBC. This plot illustrates the maximum allowable cost per scenario based on the number of months gained by the therapy (PFS). For instance, if a therapy extends one patient’s life by six months, the cost could be priced at GBP 25,000 per patient because two such regimens would prolong the life expectancy by twelve months. Therefore, extending one patient’s life by 6 months would cost GBP 25,000, and extending the life by 12 months would be equal to two patients receiving such therapy, amounting to GBP 50,000 altogether. Conversely, if an NCV regimen prolonged one patient’s life by just one month, the maximum allowable cost per patient would be GBP 4167 to stay within the NICE threshold for twelve months.

Table 9 indicates that the worst-case ICER exceeds the GBP 50,000 NICE willingness-to-pay threshold. The ICER values were plotted against this threshold (Figure 15), illustrating the maximum cost of treatment per month gained. The best-case scenario analysed in this paper falls well below the NICE threshold, accounting for only 5% of the allowable cost, indicating its high feasibility. Conversely, the worst-case ICER slightly exceeds the threshold based on the assumed PFS of 5.6 months. While the characteristics of vaccine administration are based on the relevant literature, these values are approximate estimates, and the scenarios are contingent on variables that may change. Furthermore, limitations of the worst-case scenario include the assumed costs outlined in Figure 6. Given the lack of existing NCVs on the market, the assumed R&D costs amounting to GBP 100,000,000 remain a rough estimate. Similarly, uncertainties persist regarding the estimated equipment, personnel, and facility expenses, which were derived from data on the manufacturing of an autologous DC cancer vaccine due to the absence of the relevant literature on NCVs. Thus, the analysis revealed that the best-case scenario is highly feasible, whereas the worst-case scenario, associated with greater uncertainties, exceeds the NICE threshold, implying its unfeasibility.

**Figure 16:**
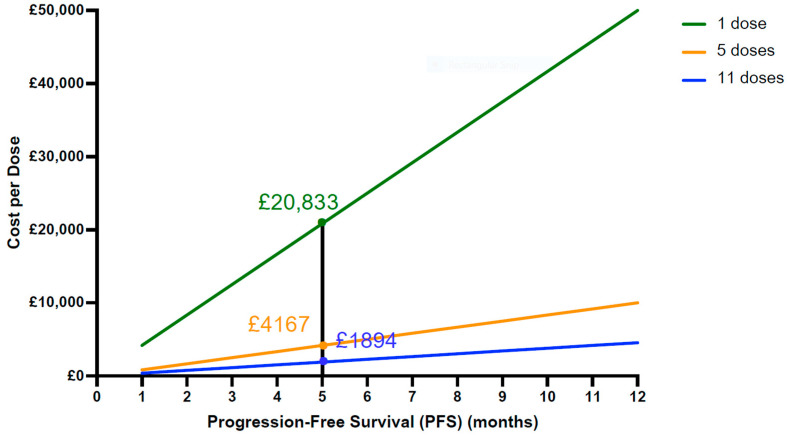
Maximum allowed cost per dose, dependent on PFS. This plot indicates that the fewer doses per regimen required, and the greater number of months gained by the regimen, the higher the potential price of a single dose can be. The black line shows the intersection between each regimen at 5 months PFS, demonstrating the maximum allowed cost per dose per scenario, respectively. The lower the number of doses per regimen, i.e., 1 dose, the greater the possibility of the cost per dose. Contrarily, the higher the number of doses per regimen, i.e., 11 doses, the smaller the value of cost per dose.

The vaccine regimen cost is, therefore, highly sensitive to two main variables: the number of doses and the PFS. Figure 16 illustrates the cost allowances across three regimen scenarios: single-dose, five-dose, and eleven-dose administration. For instance, assuming all scenarios prolong one patient’s life by 5 months, a single dose could cost up to GBP 20,833, while one dose in a five-dose regimen would be GBP 4167, and in an eleven-dose regimen, GBP 1894 per patient. Therefore, the single-dose regimen allows for the highest permissible cost per dose. This indicates that fewer doses required for the same PFS result in higher allowable costs per NCV dose. Furthermore, Figure 16 highlights the cost disparities among the regimens. Comparing the cost of doses for 5 months versus 12 months of any regimen underscores the importance of achieving a higher PFS. In the single-dose scenario, the 5-month regimen could cost GBP 20,833 per dose, while the 12-month regimen could approach the threshold of GBP 50,000. For the five-dose and eleven-dose regimens, however, the maximum allowed costs per dose would need to be considerably lower to be cost effective (Figure 16). These factors underscore the significance of minimising the number of doses and maximising each patient’s life expectancy to mitigate the risk of vaccine rejection by NICE.

The main limitations in NCV pricing include the projected PFS and low immunogenicity, necessitating multiple doses to achieve an immune response. The assumed NCV PFS used in this analysis matches chemotherapy but falls short of already established ICIs, compared with Table 8. This could impact NICE approval due to perceived insufficient benefits. However, NCVs potentially offer advantages, like reduced adverse events and broader eligibility, compared to chemotherapy and ICIs. Based on current data outlined in this review, it is, however, unlikely that NCVs could be offered as a standalone treatment due to the immunogenicity challenges. For this reason, there is currently no benefit from comparing an NCV treatment directly with the existing therapies from a financial perspective. The low immunogenicity rather suggests that further research on combination therapy with existing treatments is required. Due to the lack of papers on this topic, it can only be expected that such a strategy would increase treatment costs. Our findings suggest that R&D should prioritise improving immunogenicity to reduce required doses and enhance survival outcomes to maximise the possibility of receiving NICE approval. It is highly unlikely that an NCV for TNBC would receive approval if the vaccine efficacy, i.e., immunogenicity, does not significantly improve.

## 5. Conclusions

Neoantigen cancer vaccines (NCVs) offer a promising approach towards targeted cancer immunotherapy for triple-negative breast cancer (TNBC) treatment. The main prospect of NCVs is their feasible manufacturing process allowing for notable cost savings. However, the limited immunogenicity of NCVs is a significant challenge, and from this review, we conclude that a multi-neoantigen formulation with a toll-like receptor agonist should be further explored. The current TNBC therapy costs range between GBP 2200 and GBP 54,000, depending on the cancer stage. An opportunity for decreasing the current costs would be most impactful from stage II onwards, though it is contingent upon immune checkpoint inhibitor eligibility. It remains a question whether an NCV could alleviate some of these costs or act as an additional agent, further increasing the TNBC therapy costs. The financial feasibility of NCVs also depends on the operational setting. Calculations revealed that if the government were to manage the vaccine at cost, approval would likely be feasible. However, in a worst-case scenario where the therapy is privately owned, the cost per regimen to extend the patients’ lives exceeds the GBP 50,000 NICE approval threshold. Crucial variables influencing cost include the number of doses and progression-free survival (PFS). Analyses reveal that fewer doses and higher PFS allow for higher approved costs per dose. Therefore, clinical evaluation of dosage and PFS will be critical for further refining economic appraisal.

Further research is essential to improve immunogenicity as the main hurdle of NCVs for TNBC; recommendations in this review include exploring a multi-neoantigen setting and a combination therapy with existing treatments with the aim to enhance the currently low vaccine efficacy. Obtaining the latest country-specific data on TNBC patients and therapies is required to draw accurate conclusions about the current state of the art and to provide a reliable base for further financial evaluations ahead of the commercialisation of NCVs.

In conclusion, developing an NCV for TNBC is under current circumstances unfeasible, primarily due to immunogenicity challenges, hindering the successful development. However, the NCV cost effectiveness as a standalone factor implies that the commercialisation of the therapy is possible, and the focus should be on maximising the life prolongation factor while minimising the number of doses required per patient.

## Figures and Tables

**Table 1:** Biological definition of breast cancer stages. The prognosis of breast cancer is influenced by its stage, with advancing stages correlating with poorer outcomes. Created from Srimuninnimit et al. [11] and Pogoda et al. [12].

Stage 0	Stage I	Stage II	Stage III	Stage IV
No tumour cells spread outside the tumour	Localised tumour with some cells escaping the tumour	Lymph nodes affected	Lymph nodes, muscles, and skin affected	Tumour cells have spread to any part of the body

**Table 2:** Overview of cancer vaccine platforms. While each platform presents distinct advantages and challenges, peptide-based cancer vaccines offer practical optimisation techniques, like adjuvants.

	Peptide Based	Cell Based	Virus Based	Nucleic Acid Based
**Main Component**	Known or predicted tumour antigen epitopes [27]	DCs containing antigens [29]	Antigen encapsulated in a viral vector [30]	Tumour antigen encoded DNA/RNA [31]
**Antigen Delivery**	Tumour antigen epitopes [27]	Autologous antigen-loaded DCs [29]	Antigens in an oncolytic virus [30]	Neoantigens for DNA, TAAs and neoantigens for mRNA [31]
**Main Advantage**	Ease of production [27]	Broad antigen immune response due to containing whole tumour antigens [29]	Long-lasting immunity [30]	Simple and fast vaccine preparation [31]
**Main Disadvantage**	Low immunogenicity [28]	Expensive development [32]	Limited multi-dosing regimens [33]	Poor immunogenicity and stability [31]

**Table 3:** Proportions of treated TNBC patients. Only certain proportions of patients receive the standardised treatment, and the numbers shown are directly derived from the cited source. Subsequently, the highest percentages were extracted and are shown in this table. N.A. means that a specific therapy option is not provided to the TNBC stage patients. Created from NABCOP [35].

	Stage 0	Early Invasive TNBC	Metastatic TNBC
**Total Number of TNBC Patients per Year**	1507	12,259	608
**Surgery**	92% (1387)	89% (10,911)	N.A.
**Radiotherapy**	58%	87%	N.A.
**Chemotherapy**	N.A.	53%	25% (152)

**Table 4:** Cost values per TNBC stage. The referenced values were extracted directly from the cited sources and adjusted to January 2024 Great British Pounds. Equal distribution of early-stage TNBC (stage I–III) numbers of patients was assumed due to no other data being available in the UK.

	Stage 0	Stage I	Stage II	Stage III	Stage IV
**Cost per Patient**	GBP 2200	GBP 6800	GBP 10,000	GBP 17,500	GBP 22,000
**Original Cost Value**	USD 11,070 in 2019 [38]	USD 5167 in 2015 [36]	USD 7613 in 2015 [36]	USD 13,330 in 2015 [36]	USD 12,500 in 2014 [39]
**Number of** **Patients**	1387	3637	3637	3637	152

**Table 5:** Administration of cost specifications for atezolizumab and pembrolizumab. Atezolizumab cost was calculated by multiplying its price by 2 to obtain the cost per one cycle and was then multiplied by 6 cycles. Similarly, pembrolizumab costs GBP 2630 · 2 per dose since one dose is 200 mg, multiplied by 4 cycles. Created from NICE [40,41].

Immune Checkpoint Inhibitor	Dosage (mg)	Frequency	Price	Total Cost per Patient
Atezolizumab	840	Day 1 and 5 every 4 weeks, 6 cycles	GBP 2665.38 per 840 mg solution	GBP 32,000
Pembrolizumab	200	Once every 3 weeks, 4 cycles	GBP 2630 per 100 mg solution	GBP 21,000

**Table 6:** Number of eligible patients for atezolizumab and pembrolizumab. The number of patients per stage was adopted from Table 4. An assumed calculated 37% mean of eligible patients was assumed due to the required genetic predisposition.

Immune Checkpoint Inhibitor	TNBC Stage	Number of Patients	Eligible Patients (37%)
Atezolizumab	III and IV	3789	1402
Pembrolizumab	II and III	7274	2692

**Table 7:** Assumed regimen of the neoantigen cancer vaccine administration. A multi-neoantigen formulation in an 11-dose regimen that prolongs a patient’s life by 5.6 months was adopted from publications in the table due to the lack of late-stage trials for an NCV for TNBC.

**Number of Neoantigens in a Single Vaccine per Patient**	**5 [42]**	
**Dose of a Single Peptide in a Single Vaccine**	75 μg [43]	
**Number of Doses**	11 [44]	
**Total Amount Required of a Single Peptide per Patient**	1 mg	=75 μg · 11 doses
**Progression-Free Survival Gained**	5.6 months [45]	

**Table 8:** Incremental cost effectiveness ratio (ICER) per life year adjusted of approved TNBC therapies. Calculated cost per patient numbers are adopted from Table 5. Progression-free survival (PFS) represents the time from study initiation until tumour progression. The ICER/life year adjusted was calculated by 12 months x cost per patient/median PFS. All therapies are below the GBP 50,000 NICE threshold since the NHS received a confidential discount for atezolizumab.

	Cost per Patient	Median PFS (months)	ICER/Life Year Adjusted
**Chemotherapy**	GBP 17,500	5.6 [93]	GBP 38,000
**Atezolizumab**	GBP 32,000	7.5 [94]	GBP 52,000
**Pembrolizumab**	GBP 21,000	7.6 [95]	GBP 34,000

**Table 9:** Costs and incremental cost effectiveness ratios (ICERs) per scenario. The total cost per regimen was calculated as outlined in Table 5; best-case costs are GBP 850 + GBP 100 + GBP 125, and worst-case costs are GBP 8000 + GBP 14,000 + 2200. The ICER/life year adjusted was calculated by 12 months x total cost per regimen/5.6 PFS. The difference in ICER values stems from the decentralised approach in the best-case scenario, contrasting with the worst-case scenario where a company aims to recover its investments. While the best-case ICER is well below the GBP 50,000 NICE threshold, the worst-case scenario exceeds it.

	Best-Case Scenario	Worst-Case Scenario
**Total Cost per Regimen**	GBP 1100	GBP 25,000
**ICER**	GBP 2400	GBP 55,000

## Data Availability

The original contributions presented in this study are included in the article/Appendix A. Further inquiries can be directed to the corresponding author(s).

## Appendix A

**Table A1:** Overview of TNBC clinical trials. NA means the therapy mode was not defined in the clinical trial description. Data were accessed from clinicaltrials.gov on 22 February 2024.

NCT Number	Status	TNBC Stage	Therapy Mode	Phase	Platform
NCT03387085	Active	All stages	Immunotherapy + chemotherapy	1b/2	Viral
NCT02316457	Completed	All stages	Immunotherapy + chemotherapy	1	RNA
NCT05504707	Recruiting	Early stage	Chemotherapy	1	DCs
NCT03199040	Terminated	Advanced	Immunotherapy	1	DNA
NCT03562637	Recruiting	Early stage	Chemotherapy	3	Peptide
NCT04674306	Recruiting	Early stage	Immunotherapy	1	Peptide
NCT04634747	Not yet recruiting	Advanced	Immunotherapy + chemotherapy	2	Peptide
NCT02938442	Completed	Early stage	Chemotherapy	1/2	Peptide
NCT03362060	Active	Metastatic	Immunotherapy	1b	Peptide
NCT03674827	Terminated	Metastatic	Immunotherapy + chemotherapy	1	Unknown
NCT02593227	Completed	Advanced	Chemotherapy	2	Peptide
NCT04348747	Recruiting	All stages	Immunotherapy	2a	DCs
NCT04024800	Active	Advanced	Immunotherapy	2	Peptide
NCT02427581	Withdrawn	Advanced and metastatic	Chemotherapy	1	Peptide
NCT02348320	Completed	All stages	Chemotherapy	1	DNA
NCT04105582	Completed		Chemotherapy	1	DCs
NCT00986609	Completed	Early stage	NA	1	Peptide
NCT02826434	Active	Early stage	Immunotherapy	1	Peptide
NCT05455658	Recruiting	Early stage	NA	2	DNA
NCT04296942	Terminated	Metastatic	Immunotherapy	1	Viral
NCT02018458	Completed	Locally advanced	Chemotherapy	1/2	DCs
NCT03012100	Active	Early stage and advanced	Chemotherapy	2	Peptide
NCT00640861	Completed	Early stage	NA	1	Peptide
NCT03606967	Recruiting	Metastatic	Chemotherapy + immunotherapy	2	peptide
NCT04879888	Completed	All stages	Chemotherapy	1	DCs
NCT05329532	Recruiting	Advanced	Immunotherapy	1/2	Peptide

**Table A2:** Conventional therapy costs per TNBC stage. The patient count was calculated from NABCOP [35], and per-patient costs were derived from research papers. The cost per stage increases with more advanced stages, resulting in a GBP 130,000,000 annual cost for conventional TNBC therapy in the UK.

	Stage 0	Stage I	Stage II	Stage III	Stage IV
**Number of Patients**	1387	3637	3637	3637	152
**Cost per Patient**	GBP 2200	GBP 6800	GBP 10,000	GBP 17,500	GBP 22,000
**Total Yearly Cost per Stage**	GBP 3,100,000	GBP 25,000,000	GBP 37,000,000	GBP 64,000,000	GBP 3,400,000

**Table A3:** Adjusted total annual costs of atezolizumab and pembrolizumab for TNBC. Atezolizumab is for stage III and IV TNBC, while pembrolizumab is for stage II and III TNBC. A 37% eligibility assumption due to genetic predisposition was made.

Immune Checkpoint Inhibitor	Eligible Patients (37%)	Cost per Patient	Total Cost
Atezolizumab	1402	GBP 32,000	**GBP 45,000,000**
Pembrolizumab	2692	GBP 21,000	**GBP 57,000,000**

**Table A4:** Adjusted number of patients eligible for immune checkpoint inhibitor (ICI) therapy. If 50% of TNBC patients exhibited the required genetic predisposition, the costs per ICI would rise significantly in the UK.

Immune Checkpoint Inhibitor	Number of Patients	Eligible Patients (50%)	Cost per Patient	Adjusted Total Cost
Atezolizumab	3789	1895	GBP 32,000	GBP 61,000,000
Pembrolizumab	7274	3637	GBP 21,000	GBP 77,000,000
